# A Treatise on Fractures

**Published:** 1883-04

**Authors:** 


**A Treatise on Fractures.** By LEWIS A. STIMSON, B. A., M. D., Professor of Surgical Pathology in the Medical Faculty of the University of New York; Attending Surgeon to the Bellevue and Presbyterian Hospitals, New York. With 360 illustrations. Philadelphia: H. C. Lea's Son & Co. 1883. Octavo, 600 pages. Price, $4.75.

This book is offered to the profession on its merits without a preface, the usual apology from the author for venturing to appear in print. After a careful examination of it we are able to heartily congratulate the author on the success of his work. It is indeed a valuable and comprehensive addition to the surgical literature of the profession. We predict that it will take rank as one of the best treatises on fractures in the English language. The author is fortunate in the selection of his publisher. The engravings are new; and, like all of H. C. Lea's books, the press-work is excellent and the volume comes out in a handsome and substantial dress.

